# Development of a Hybrid Course on Wheelchair Service Provision for clinicians in international contexts

**DOI:** 10.1371/journal.pone.0199251

**Published:** 2018-06-15

**Authors:** Yohali Burrola-Mendez, Mary Goldberg, Rachel Gartz, Jon Pearlman

**Affiliations:** 1 Department of Rehabilitation Science and Technology, University of Pittsburgh, Pittsburgh, Pennsylvania, United States of America; 2 International Society of Wheelchair Professionals (ISWP), University of Pittsburgh, Pittsburgh, Pennsylvania, United States of America; Universita degli Studi di Perugia, ITALY

## Abstract

**Introduction:**

Wheelchair users worldwide are at high risk of developing secondary health conditions and premature death due to inappropriate wheelchair provision by untrained providers. The International Society of Wheelchair Professionals (ISWP) has developed a Hybrid Course based on the World Health Organization’s Wheelchair Service Training Package—Basic Level. The Hybrid Course leverages online modules designed for low-bandwidth internet access that reduces the in-person training exposure from five to three and a half days, making it less expensive and more convenient for both trainees and trainers.

**Methods:**

The Hybrid Course was designed using a systematic approach guided by an international group of stakeholders. The development followed the Quality Matters Higher Educational Rubric, web design guidelines for low bandwidth, experts’ opinions, and the best practices for blended course design. A quasi-experimental approach was used to evaluate the effectiveness of the Hybrid Course taken by six graduate students in Rehabilitation Sciences at the University of Pittsburgh by measuring pre- and post knowledge using the validated ISWP Wheelchair Service Provision—Basic Test. The outcome measure was assessed using a paired sample t-test between pretest and posttest scores. The quality of the Hybrid Course was evaluated by three external reviewers using the Quality Matters Higher Educational Rubric who were blind to each others’ evaluation and the results of the training intervention.

**Results:**

Hybrid Course participants reported significant increases in scores on the ISWP Wheelchair Service Provision—Basic Test after participating in the training, with an average increase of 10.84±5.42, p = 0.004, Cohen’s *d* = 1.99. In addition, the Hybrid Course met the Quality Matters Standards in two out of three evaluations and reported a percentage of agreement between evaluators of 84%.

**Conclusions:**

The Hybrid Course met quality standards and proved to be effective in increasing basic level wheelchair knowledge in a group of Rehabilitation Science graduate students.

## Introduction

The World Health Organization (WHO) estimates that 10% of people with disabilities, approximately 112 million people, need a wheelchair for mobility and function. However, only 5%–15% of them have access to a properly fitted wheelchair, indicating that approximately 96 million people do not have a wheelchair or have one that does not meet their needs [1–3]. Wheelchair users who do not have access to appropriate wheelchair provision by trained providers are at a high risk of developing secondary health conditions and premature death [1, 4]. The lack of training of personnel delivering wheelchair services may result in poorly fitted wheelchairs that are difficult to propel, fail prematurely and cause injuries to the user [1, 4, 5]. The health complications of inappropriate wheelchair provision include pressure injuries, falls, overuse or repetitive strain injuries, postural deformities, restricted breathing, and a limited range of motion [1, 6]. This situation suggests that countries where inappropriate wheelchair service delivery is occurring are not fulfilling the promise of the United Nations Convention on the Rights of People with Disabilities (UNCRPD), which entitles all people to the right of personal mobility [7].

In 2008 the WHO, the United States Agency for International Development (USAID), the International Society for Prosthetics and Orthotics, and Disabled Peoples’ International launched the Guidelines for the Provision of Manual Wheelchairs in Less-Resourced Settings as an international effort to promote training and to assist nations in fulfilling the United Nations Convention on the Rights of People with Disabilities (UNCRPD) [1]. The WHO Guidelines outline eight service steps and the minimum standards that form the basis of a comprehensive wheelchair service based on international evidence-based practice and research [1, 6, 8, 9]. Following the release of the WHO Guidelines, the WHO and USAID, published a series of Wheelchair Service Training Packages (WHO WSTPs) to support clinicians’ training and to increase wheelchair access worldwide. To date, there are five WHO WSTPs: Basic, intermediate, managers’, and stakeholders’, and trainers’ packages [10–14]. All of the WHO WSTPs follow a learning methodology of in-person training held over consecutive days. This training format may make it difficult for busy providers to attend and to scale across multiple settings, including university training programs. As a result, there still is a widespread delivery of inappropriate wheelchairs worldwide, indicating that the training uptake has been slow, and capacity is insufficient.

Blended learning, or hybrid learning, is a mix of different learning environments and approaches that include online and in-person methods [15]. This type of learning is a cost-effective [16] and student-accepted [17–19] method of knowledge dissemination and could feasible for global health education [20–22]. Literature has demonstrated that blended learning is as effective as in-person learning in medical and non-medical education [15, 17, 23–25] and can be a feasible solution to overcome knowledge dissemination barriers in less-resourced areas [20].

As blended education proliferates, so do efforts to evaluate its effectiveness by research and assessment groups worldwide such as the International Association for K-12 Online Learning (iNACOL) [26], Education Elements [27], the University of Central Florida—Blended Learning Toolkit [28], Quality Matters [29], and Educause [30], among others. One such organization, Quality Matters (QM), is an international leader in rubric development and quality assurance for online education. QM has developed rubrics intended to guide the development, evaluation, and improvement of online and blended courses, such as the QM Higher Education Rubric [29, 31]. This rubric includes 8 General Standards and 43 Specific Review Standards used to evaluate the design of online and blended courses [29, 31, 32]. To certify the quality of a course, QM requires at least 85% of the content to meet the quality expectations of the rubrics. Multiple studies have used the QM Rubrics successfully to guide the development and to assess the quality of online and blended courses [33–37].

Motivated by the successful learning outcomes using blended courses as described in the literature and with the aim of offering alternative learning methodologies that increase the spread of wheelchair service delivery training, our goal was to develop and evaluate a blended learning approach for the WHO WSTP-Basic level (WHO WSTP-B).

The specific aims of this action research study were to:

1. Determine the online design criteria and content allocation and develop online modules in English.
2. Do a pilot test of the Hybrid Course to evaluate the learning effect.
3. Evaluate the quality of the Hybrid Course using the Quality Matters Higher Education Rubric.

We hypothesized that trainees of the Hybrid Course would have significantly higher scores on the ISWP Wheelchair Service Provision–Basic Test after receiving training and that the Hybrid Course would receive an adequate rubric score (85%) to meet the quality standards for blended courses.

## Materials and methods

Each specific aim was completed in sequence; the methods are described below.

### Specific aim 1: Determine the online design criteria, the allocation of online content, and develop online modules in English

#### Identify the online design criteria

**Fig 1:**
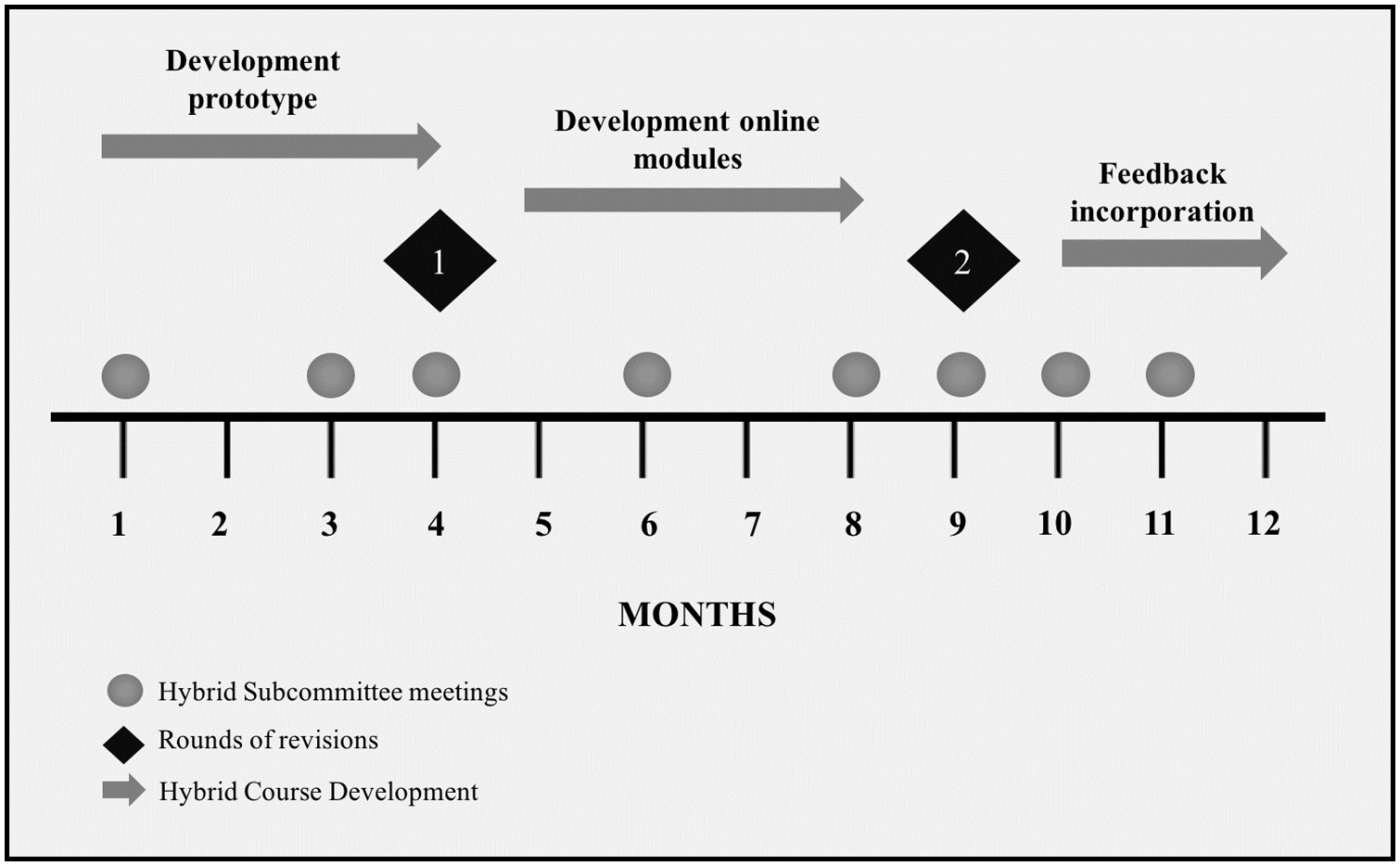
Overview of the development of the Hybrid Course.

To address **specific aim 1** the International Society of Wheelchair Professionals (ISWP, a coordinating body for the wheelchair sector) [38] formed the Hybrid Subcommittee (HSC), a multidisciplinary and international stakeholders group that guided the development of the Hybrid Course. The HSC was represented by eight members from high-, middle- and low-income countries (Brazil, Canada, Colombia, India, Mexico, Philippines, the United Kingdom, and the United States of America) with experience in delivering wheelchair training and developing educational programs for high- and low-resource settings. Over the course of 12 months (May 2015 to April 2016), the HSC held synchronous monthly online meetings to discuss and advise upon the development of the Hybrid Course (Fig 1). ISWP staff attended the meetings to help coordinate the sessions and distribute the agenda and the minutes. All meetings were recorded and made available to HSC members [39]. An ISWP core team member and HSC member (YBM) with both curriculum and course development and clinical wheelchair experience was the primary developer of the Hybrid Course with advice from the co-authors of this study and the HSC.

#### Identify design criteria

The HSC considered the QM Higher Education Rubric [32] to be a useful framework to guide the development of the Hybrid Course. However, the HSC identified that the Rubric lacks strategies to implement courses in international contexts where connectivity and low internet speeds may be challenging. The HSC reviewed the best practices on blended course design [40] and considered its members’ experience developing educational programs and delivering training in international settings to offer suggestions that strengthened the QM Higher Education Rubric and guided the development of online modules.

#### Determine the appropriate allocation of online content

The aim of the Hybrid Course is to offer an alternative learning methodology for the WHO WSTP-B. The purpose of the WHO WSTP-B is to develop the skills and knowledge of personnel who are required to deliver basic level wheelchair services to people with mobility impairments who can sit upright without additional postural support [10]. No clinical background is required to access the training, which makes it feasible to replicate in places where there are few or no professionals in the field of seating and mobility [10]. Table 1 presents the content and time allocation of the WHO WSTP-B in its original learning methodology of in-person training.

**Table 1:** WHO WSTP-B content and time allocation.

Day 1	Day 2	Day 3	Day 4	Day 5
**Introduction**	A.6 Appropriate wheelchair (continue)	B.4 Physical assessment (continue)	B.9 Fitting	Practical Three
**A. Core Knowledge**	A.7 Cushions	B.5 Prescription (selection)	B.10 Problem solving	Practical Four
A.1 Wheelchair users	A.8 Transfers	B.6 Funding and ordering	B.11 User training	B.14 Putting it all together
A.2 Wheelchair services	**B. Wheelchair Service Steps**	Practical One	B.12 Maintenance and repairs	Presentation of certificates
A.3 Wheelchair mobility	B.1 Referral and appointment	B.7 Product (wheelchair) preparation	Practical Two	
A.4 Sitting upright	B.2 Assessment	B.8 Cushion fabrication	B.13 Follow up	
A.5 Pressure sores	B.3 Assessment interview			
A.6 Appropriate wheelchair	B.4 Physical assessment			

Adapted from World Health Organization, 2012 [10]

The HSC and the primary developer of the Hybrid Course analyzed the theoretical and practical components of the WHO WSTP-B to select the most appropriate content to host online.

#### Online module development

The primary developer of the Hybrid Course (YBM) utilized the QM Higher Education Rubric [32], HSC recommendations, web design guidelines for low bandwidth [41] and best practices for blended course design [40] to develop a set of specific review standards that guided the development of the Hybrid Course. The development of the online modules included two rounds of internal (co-authors) and external (HSC) revisions (Fig 1). In the first round, a module prototype was developed and distributed to the HSC members to collect feedback about the visual design of the course, the modules’ sections and the layouts. In the second round of revision, all modules and their respective content were created and distributed via the online platform. For this round, feedback was solicited on curriculum and platform access. In terms of curriculum, reviewers were asked to verify that the content (learning objective, topics, quizzes, and activities) strictly followed the WHO WSTP course. To evaluate the platform access, HSC members were asked to test the modules in different settings with high and low internet connection speeds and using different technology devices such as smartphones, tablets, laptops, and desktop computers.

### Specific aim 2: Do a pilot test of the Hybrid Course

To address **specific aim 2**, a quasi-experimental trial utilized a pretest-posttest design to evaluate changes in basic level wheelchair knowledge using the validated ISWP Wheelchair Service Provision–Basic Test [42]. The study was approved by the University of Pittsburgh Institutional Review Board.

#### Study sample

**Fig 2:**
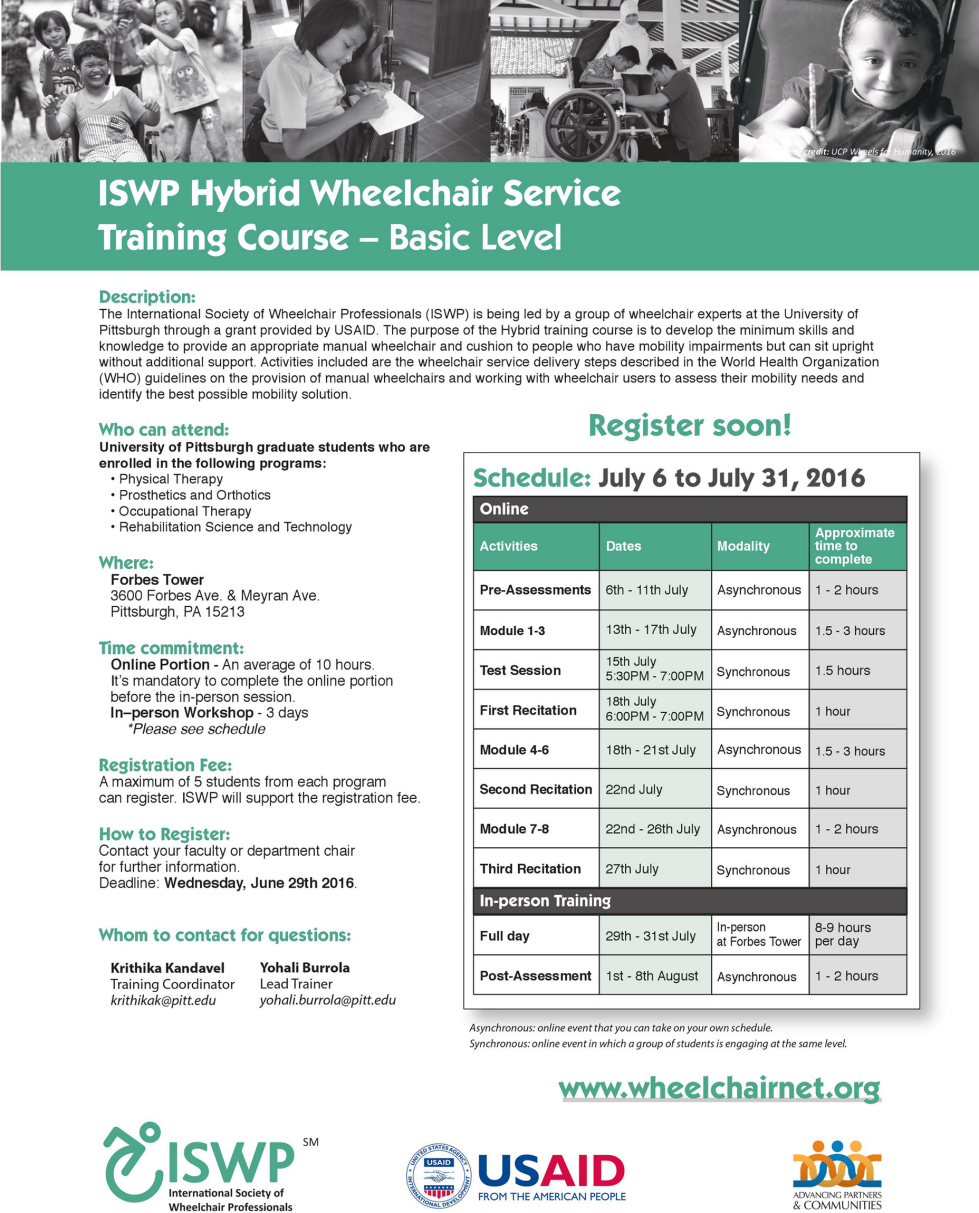
The Hybrid Course flyer.

The sample was selected using a convenience sampling method guided by the co-authors and the HSC. The research team met with the academic directors of the Physical Therapy (PT), Occupational Therapy (OT), Prosthetics and Orthotics (P&O), and Rehabilitation Science and Technology (RST) programs from the University of Pittsburgh to inform and share the scope of the project. The academic directors distributed the Hybrid Course flyer to their students inviting them to register for the Hybrid Course. The flyer included the description of the course, inclusion criteria, location, online and in-person time commitments, schedule, registration process and contact information (Fig 2).

The study’s inclusion criteria included: 1) students, staff, or professors from the PT, OT, P&O, and RST programs from the University of Pittsburgh; 2) who have not taken the ISWP Wheelchair Service Provision–Basic Test. We excluded participants who were simultaneously working on other wheelchair-related study or training.

#### Outcome measure: Wheelchair Service Provision knowledge

The ISWP Wheelchair Service Provision–Basic Test is a valid method for measuring the basic competency of wheelchair professionals independent of geographic location[42]. The test consists of 19 sociodemographic questions and 75 multiple-choice questions that evaluate basic wheelchair service delivery. The multiple-choice questions evaluate seven domains of wheelchair service delivery: 1) assessment, 2) prescription, 3) fitting, 4) production, 5) user training, 6) process, and 7) follow-up and maintenance as covered in the WHO WSTP-B. The domains have different weights based on the pre-set number of questions that each domain was allocated. Each domain has a pool of questions created to reduce the likelihood of receiving the same question when taking the test multiple times. Test scores greater than or equal to 53 points (70% of the total points) are considered passing scores. The test was hosted and distributed online through the testing platform, Test.com^®^.

#### Intervention

**Fig 3:**
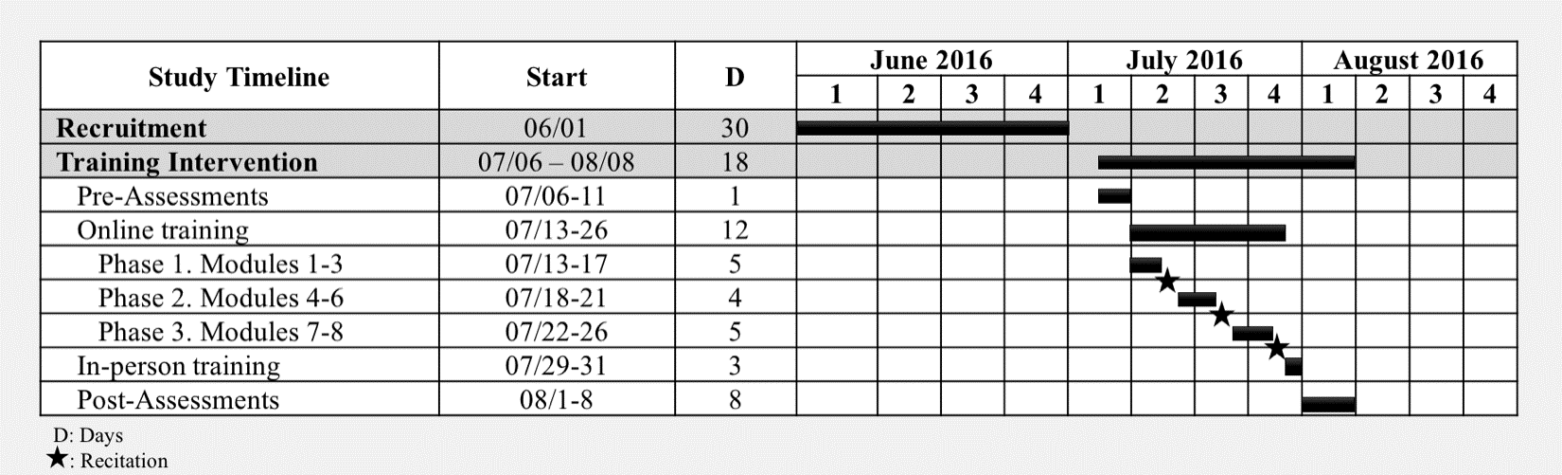
Study’s overview and timeline.

Fig 3 presents the study’s overview and timeline. The training was purposefully implemented interprofessionally. Two trainers were physical therapists and one was an occupational therapist. The trainers had been trained in the WHO WSTP-B and have participated in the WHO Wheelchair Service Training of Trainers Package–Basic level [14]. In addition, all the trainers have facilitated the WHO WSTP-B and have provided basic level wheelchair services in both high- and less-resource settings.

Assessments were collected one-week before and after the training intervention. The participants received an email with the instructions on how to log into the testing platform, Test.com^®^, and the contact information of ISWP staff in case of technical problems or questions. Participants were instructed to complete the test without accessing course materials. The settings of ISWP Wheelchair Service Provision–Basic Test included: 1) a random distribution of questions and answers from the domains’ pool of questions; 2) forced completion that required the participants to complete the test in a one-time entry; and 3) feedback and scores where the test provided the immediate test scores and the option to review correct and incorrect answers.

**Online training**: As indicated in Fig 3, the online learning was divided into three sequential phases. At each phase, the participants reviewed the content and completed the required activities asynchronously. After the completion of each phase, an online session (recitation) occurred synchronously between the participants and the trainers. During the recitations, trainers reinforced the key points of the modules, answered questions, discussed topics and promoted interactions between the participants. The recitations were recorded and made available to the participants and trainers. ISWP staff helped to coordinate the recitations and provide support if needed. Table 2 presents the training agenda of the online and in-person sessions.

**Table 2:** Online and in-person training agenda.

Online Modules			In-person sessions		
Phase 1	Phase 2	Phase 3	Day 1	Day 2	Day 3
Introduction	A.4 Sitting upright	A.7 Cushions	Welcome, introductions and housekeeping	B.6 Funding and ordering	Practical 1–4: All steps
A.1 Wheelchair users	A.5 Pressure sores	A.8 Transfers	Practical 1: Assessment and Prescription	B.7 Product (wheelchair) preparation	Closing, certificates
A.2 Wheelchair services	A.6 Appropriate wheelchair		A.5 Pressure sores (practicals)	B.8 Cushion fabrication	
A.3 Wheelchair mobility			A.7 Cushions (practicals)	B.9 Fitting	
			A.8 Transfers (practicals)	B.10 Problem solving	
			B.1 Referral and appointment	B.11 User training	
			B.2 Assessment	B.12 Maintenance and repairs	
			B.3 Assessment interview		
			B.4 Physical assessment		
			B.5 Prescription (selection)		

**In-person training**: After the completion of the online modules, the participants attended three days of in-person training led by the three trainers at the University of Pittsburgh, USA (Table 2). Three experienced wheelchair users were also invited to participate as volunteers in the in-person sessions, and the trainees had the opportunity to work directly with them throughout the in-person sessions.

#### Data management and analysis

All data was collected in a Test.com^®^ database, exported into a CSV file and then into SPSS^®^ Version 24.0. Descriptive statistics were calculated. For the outcome measure, knowledge change, a paired sample t-test was calculated to compare the levels of knowledge between the baseline and post-training total scores. In addition, paired sample t-tests were calculated for each test domain to explore specific knowledge changes. The effect size was calculated using Cohen’s *d* [43]. All analyses were carried out using an exploratory alpha level of 0.05.

### Specific aim 3: Evaluate the quality of the Hybrid Course using the Quality Matters Higher Education Rubric

To address **specific aim 3**, ISWP recruited three evaluators from two middle-income countries, fluent in English, with experience implementing educational health programs in high- and less-resourced settings using in-person and blended learning methodologies. This quality evaluation was conducted after the implementation of the Hybrid Course.

The QM Higher Education Rubric was used to evaluate the quality of the Hybrid Course. This tool includes 8 General Standards: 1) Course Overview and Introduction; 2) Learning Objectives (Competencies); 3) Assessment and Measurement; 4) Instructional Materials; 5) Course Activities and Learner Interaction; 6) Course Technology; 7) Learner Support; and 8) Accessibility and Usability; and 43 Specific Standards that are dichotomously rated as either “met” or “not met” [31, 32]. When a Specific Standard is met, it receives a pre-assigned value of 1, 2, or 3 points; those with point values of 3 are considered “essential standards” and must be met for a course to “meet standards” [32, 44]. The maximum score of the Rubric is 99 points. A score of 85 points out of 99, or 85%, as well as meeting all 3-point essential standards, is required for a course to meet the QM Standards.

The evaluators received an individual invitation via email from the primary developer of the Hybrid Course to voluntarily review and evaluate the course using the QM Higher Education Rubric. Upon agreeing to participate, a second email was sent with the link and instructions to access and review the Hybrid Course and the QM Higher Education Rubric attached. Reviewers had six weeks to evaluate the Hybrid Course and return the rated QM Rubric via email. In addition, they were encouraged to submit suggestions and comments to help to improve the course and to contact the author with any inquiries. Reviewers were blinded to both each other’s evaluations and the pilot results from the Hybrid Course.

The rated rubrics were transcribed to a Microsoft Excel spreadsheet database. A total score was obtained per reviewer by adding all the pre-assigned value (1, 2, or 3 points) of the Specific Standards that were met. In addition, the percentage of agreement was computed by calculating the number of times raters agreed on a rating and then divided by the total number of ratings [45, 46]. This technique allows exploration of agreement of multiple evaluators and the opportunity to identify items that may be problematic [46]. An odd number of reviewers was set to identify probably problematic items. The HSC and the co-authors of the study determined that if an item was rated as “not met” by two or more reviewers, it would be considered as problematic. Addressing problematic items will be part of future studies. The additional recommendations offered by the HSC were included in the rubric. Reviewers were asked to evaluate if the items suggested by the HSC were “met” or “not met”; however, these items did not receive any points to not interfere with the QM’s review process.

## Results

### Specific aim 1: Determine the online design criteria, the allocation of online content, and develop online modules in English

#### Identify the online design criteria

The HSC offered eight suggestions to strengthen the QM Higher Education Rubric and to guide the development of the online modules. The suggestions were added to the original QM Rubric and are presented in Table 3. The items 1.10, 1.11, 3.6, 3.7, 5.5, 8.6, 8.7, and 8.8 represent HSC’s suggestions. The category *Accessibility and Usability* received the most suggestions (8.6, 8.7, 8.8), reflecting HSC’s emphasis on developing a course that could be shared and scaled in the future. To meet the accessibility and usability requirements, the authors selected Adobe Captivate 9^®^ and CourseSites by Blackboard^®^ as the authoring tool and the learning management system, respectively, to develop and host the online modules of the Hybrid Course. The selection was made based on the availability of the Adobe Captivate 9^®^ program and the free hosting and publishing online courses that CourSesites^®^ offers [47].

**Table 3:** Test results from the Quality Matters Higher Education Rubric and the additional Hybrid Subcommittee items.

General Standards	Specific Standards	Evaluators		
		1	2	3
**Course Overview and Introduction**	1.1 Instructions make clear how to get started and where to find various course components. (3 points)	3	3	3
	1.2 Learners are introduced to the purpose and structure of the course. (3 points)	3	3	3
	1.3 Etiquette expectations (sometimes called “netiquette”) for online discussions, email, and other forms of communication are clearly stated. (2 points)	2		2
	1.4 Course and/or institutional policies with which the learner is expected to comply are clearly stated, or a link to current policies is provided. (2 points)	2	2	2
	1.5 Minimum technology requirements are clearly stated and instructions for use provided. (2 points)	2	2	2
	1.6 Prerequisite knowledge in the discipline and/or any required competencies are clearly stated. (1 point)	1	1	1
	1.7 Minimum technical skills expected of the learner are clearly stated. (1 point)	1	1	1
	1.8 The self-introduction by the instructor is appropriate and is available online. (1 point)	1	1	1
	1.9 Learners are asked to introduce themselves to the class. (1 point)	1	1	1
	*1*.*10 The course should strictly follow the structure and content of the WHO WSTP-B*	x	x	x
	*1*.*11 The Introduction and Core Knowledge are allocated online while the Wheelchair Service Basic Steps’ section is the face-to-face component*	x	x	x
*Subtotal*		*16*	*14*	*16*
**Learning Objectives**	2.1 The course learning objectives, or course/program competencies, describe outcomes that are measurable. (3 points)	3	3	3
	2.2 The module/unit learning objectives or competencies describe outcomes that are measurable and consistent with the course-level objectives or competencies. (3 points)	3	3	3
	2.3 All learning objectives or competencies are stated clearly and written from the learner’s perspective. (3 points)	3	3	3
	2.4 The relationship between learning objectives or competencies and course activities is clearly stated. (3 points)	3		3
	2.5 The learning objectives or competencies are suited to the level of the course. (3 points)	3	3	3
*Subtotal*		*15*	*12*	*15*
**Assessment and Measurement**	3.1 The assessments measure the stated learning objectives or competencies. (3 points)	3	3	3
	3.2 The course grading policy is stated clearly. (3 points)	3	3	3
	3.3 Specific and descriptive criteria are provided for the evaluation of learners’ work and are tied to the course grading policy. (3 points)	3		3
	3.4 The assessment instruments selected are sequenced, varied, and suited to the learner work being assessed. (2 points)	2	2	2
	3.5 The course provides learners with multiple opportunities to track their learning progress. (2 points)	2	2	2
	*3*.*6 The assessments and activities accommodate different types of learners*.	x	x	
	*3*.*7 The course provides assessment strategies that include prompt and specific feedback*.	x		x
*Subtotal*		*13*	*10*	*13*
**Instructional Materials**	4.1 The instructional materials contribute to the achievement of the stated course and module/unit learning objectives or competencies. (3 points)	3	3	3
	4.2 Both the purpose of instructional materials and how the materials are to be used for learning activities are clearly explained. (3 points)	3	3	3
	4.3 All instructional materials used in the course are appropriately cited. (2 points)	2	2	2
	4.4 The instructional materials are current. (2 points)	2	2	2
	4.5 A variety of instructional materials is used in the course. (2 points)	2	2	
	4.6 The distinction between required and optional materials is clearly explained. (1 point)	1		1
*Subtotal*		*13*	*12*	*11*
**Course Activities and Learner Interaction and Engagement**	5.1 The learning activities promote the achievement of the stated learning objectives or competencies. (3 points)	3	3	3
	5.2 Learning activities provide opportunities for interaction that support active learning. (3 points)	3	3	3
	5.3 The instructor’s plan for classroom response time and feedback on assignments is clearly stated. (3 points)	3	3	3
	5.4 The requirements for learner interaction are clearly stated. (2 points)	2	2	
	*5*.*5 The course includes asynchronous and synchronous activities*.	x		x
*Subtotal*		*11*	*11*	*9*
**Course Technology**	6.1 The tools used in the course support the learning objectives and competencies. (3 points)	3	3	3
	6.2 Course tools promote learner engagement and active learning. (3 points)	3		3
	6.3 Technologies required in the course are readily obtainable. (2 points)	2	2	2
	6.4 The course technologies are current. (1 point)	1	1	1
	6.5 Links are provided to privacy policies for all external tools required in the course. (1 point)	1	1	1
*Subtotal*		*10*	*7*	*10*
**Learner Support**	7.1 The course instructions articulate or link to a clear description of the technical support offered and how to obtain it. (3 points)	3	3	3
	7.2 Course instructions articulate or link to the institution’s accessibility policies and services. (3 points)	3	3	3
	7.3 Course instructions articulate or link to an explanation of how the institution’s academic support services and resources can help learners succeed in the course and how learners can obtain them. (2 points)	2	2	
	7.4 Course instructions articulate or link to an explanation of how the institution’s student services and resources can help learners succeed and how learners can obtain them. (1 point)	1		
*Subtotal*		*9*	*8*	*6*
**Accessibility and Usability**	8.1 Course navigation facilitates ease of use. (3 points)	3	3	3
	8.2 Information is provided about the accessibility of all technologies required in the course. (3 points)	3	3	3
	8.3 The course provides alternative means of access to course materials in formats that meet the needs of diverse learners. (2 points)	2	2	2
	8.4 The course design facilitates readability. (2 points)	2		2
	8.5 Course multimedia facilitate ease of use. (2 points)	2	2	2
	*8*.*6 The course was developed considering low bandwidth requirements*			x
	*8*.*7 The course is compatible with SCORM and LMS*	x	x	x
	*8*.*8 The course is hosted in a platform that allows mobile content access*.	x	x	x
*Subtotal*		*12*	*10*	*12*
***TOTAL***		**99**	**84**	**92**

Hybrid Subcommittee’s suggestions are highlighted by the grey panels

“x” indicates the item was met

#### Determine the appropriate allocation of online content

The HSC reached a consensus and selected the Introduction and Core Knowledge to be the online components of the Hybrid Course due to their theoretical components and the few practical activities that they included (Table 4). The Wheelchair Service Basic Steps section was selected to be the in-person component of the Hybrid Course following the methodology proposed by the WHO WSTP-B. Table 5 presents the Hybrid Course content distribution.

**Table 4:** WHO WSTP-B time allocation for practicals sessions.

Section	Modules	Total time allocation (min)	Total time allocation (min) for practicals	Percentage of the content allocated for skills practicals (%)
1. Introduction		60	0	0
2. Core knowledge	A.1–A.8	600	155	25.83
3. Wheelchair Service Steps	B.1–B.14 and practicals	1545	905	58.57

**Table 5:** Hybrid Coturse content distribution.

Learning Modality	Section	Topics
**Online**	**Introduction**	
	**A. Core Knowledge**	A.1 Wheelchair users
		A.2 Wheelchair services
		A.3 Wheelchair mobility
		A.4 Sitting upright
		A.5 Pressure sores
		A.6 Appropriate wheelchair
		A.7 Cushions
		A.8 Transfers
**In-person**	**B. Wheelchair Service Steps**	B.1 Referral and appointment
		B.2 Assessment
		B.3 Assessment interview
		B.4 Physical assessment
		B.5 Prescription (selection)
		B.6 Funding and ordering
		Practical One
		B.7 Product (wheelchair) preparation
		B.8 Cushion fabrication
		B.9 Fitting
		B.10 Problem solving
		B.11 User training
		B.12 Maintenance and repairs
		Practical Two
		B.13 Follow up
		Practical Three
		Practical Four
		B.14 Putting it all together
		Presentation of certificates

#### Develop the online modules

The specific actions implemented by the primary author to fulfill the QM Rubric and the additional HSC suggestions are grouped based on the QM’s Standards and described below.

**Fig 4:**
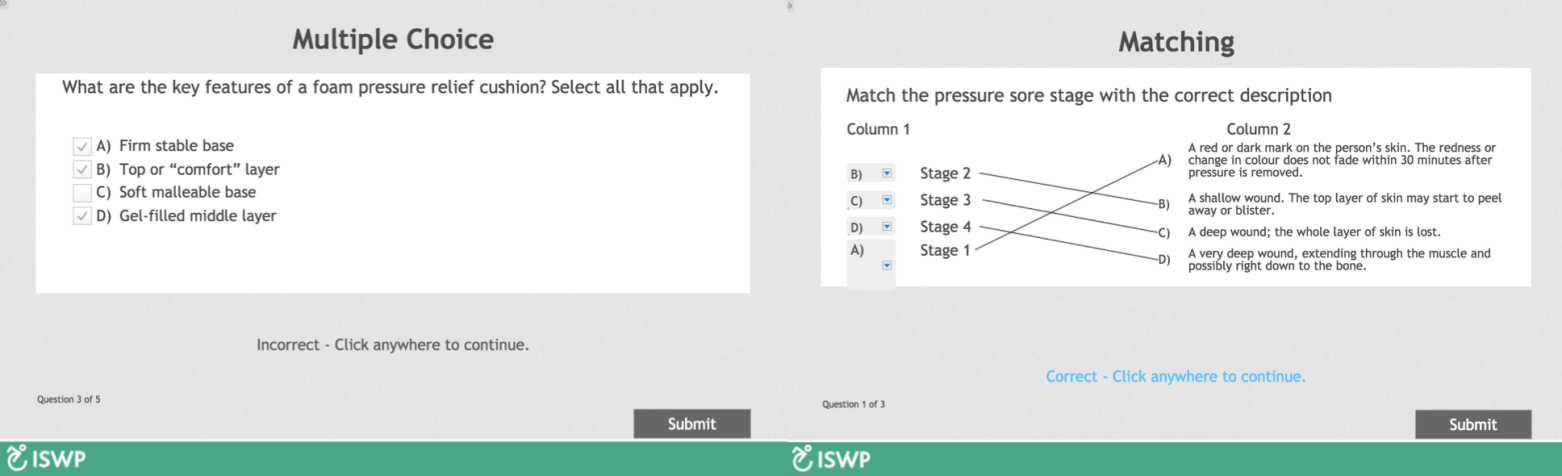
Examples of questions automatically evaluated.

**Fig 5:**
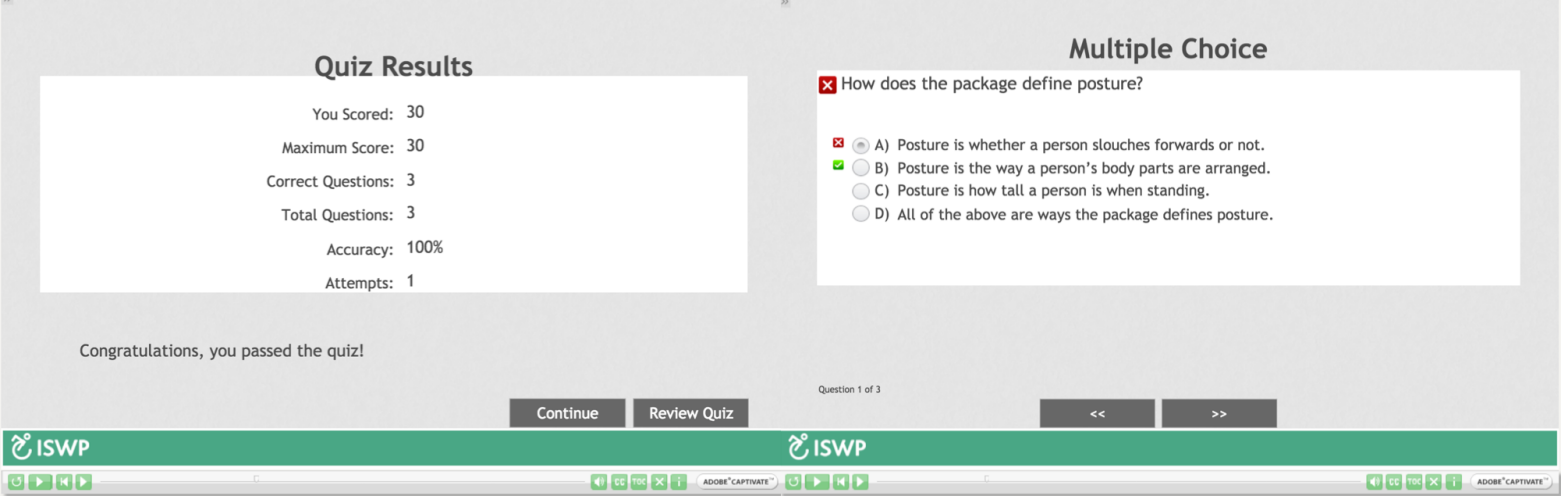
Quiz results and individual questions feedback.

1. **Course Overview and Introduction**: An Introductory module was developed that included: 1) an overview of the Hybrid Course that covered structure of the course, the purpose of the course, target audience, prerequisites, instructional materials, technology requirements, instructional videos explaining how to access CourseSites^®^ and how to navigate through the Adobe Captivate^®^ modules; and 2) tips on how to succeed in online learning. This module was hosted at ISWP’s website and distributed via an external link to the participants prior to the beginning of the course.
2. **Learning Objectives**: Each online module of the Core Knowledge included learning objectives according to the WHO WSTP-B.
3. **Assessment and Measurements**: Short quizzes (three to five questions) were developed to monitor the participants’ comprehension of the material. They were developed by the primary author and reviewed by the co-authors and the HSC. The quizzes included different types of questions such as multiple choice, multiple answers, matching columns, case studies, and true or false (Fig 4). The quizzes were automatically evaluated; this allowed the participants to receive feedback and to review the quiz immediately after its completion. Passing scores were not required to continue with the in-person training; however, all modules with their respective quiz needed to be completed. Individual scores were automatically reflected in the grading center allowing the participants and trainers to track the learning progress (Fig 5).
4. **Instructional Materials**: An electronic version of the two required materials, the WHO Reference Manual for Participants and the Participant’s Workbook, were available for participants to download from CourseSites^®^ prior to the training. Moreover, if the participants preferred to read the materials online, each module included the reading or activity that needed to be reviewed. This feature centralized resources and improved navigation throughout the course. Some modules included a folder with optional reading materials.
5. **Course Activities and Learner Interaction**: Course Activities: The online modules followed all the activities suggested by the WHO Trainer’s Manual–Basic Level; however, 25.8% of the allocated time to Core Knowledge is used to practice skills (Table 3). In order to comply with the WHO WSTP-B content structure, the practical activities were allocated to the first day of the in-person portion. The online modules introduced the activity with a video; after watching it, the participants were informed that they will practice those skills during the in-person portion of the training.
  Learner Interaction: With the aim of supporting active learning and promoting participants’ and trainers’ interaction, two activities were developed: 1) Discussion Forums, in which the participants were asked to post questions for each module and encouraged to respond to each others’ inquiries. Trainers monitored the discussion forums and added necessary information. This feature promoted human interaction consisting of two-way communication between one student to another student and between students and trainers; and 2) Recitations, three synchronous recitations were conducted to monitor the online section; the first, after the completion of the first three modules; the second, after the completion of the next three modules; and the third, after completing the last two modules (Fig 3). During the recitations, facilitators reinforced the key elements of each module and responded to participants’ questions that were not addressed by their peers.
6. **Course Technology**: The Introductory module included a section about technology requirements needed to access CourseSites^®^, where the online modules are hosted. This section has a link to a browser checker that verifies whether CourseSites^®^ supports a learner’s browser and operating system [48] and provided feedback on what steps to follow to be able to view content within CourseSites^®^.
7. **Learner Support**: The Introductory module included a section on how to succeed in online learning, that described effective communication skills and how to get technical support during the online components of the training. The participants could send emails to the trainers and primary developer of the modules through CourseSites^®^. In addition, the platform includes a help center with information about common issues and student FAQs [49].
8. **Accessibility and Usability**: Adobe Captivate 9^®^ and CourseSites by Blackboard^®^ were selected as the authoring tool and the learning management system to develop and host the online modules. The tools are compatible with Sharable Content Object Reference Model (SCORM) and Learning Management System (LMS) criteria suggested by the HSC to share and scale the training program [50, 51]. Also, Adobe Captivate^®^ and CourseSites^®^ met the low bandwidth requirements and mobile content accessibility needed to implement training programs in settings with slow internet connection speeds [47, 52].

### Specific aim 2: Do a pilot test of the Hybrid Course

A total of six participants were recruited; all of them completed the pre-and post-assessments, and therefore there were no dropouts. The average age was 26±3 years. The participants’ characteristics are described in Table 6. The demographic questions that referred to the work setting, age group served, and motivation to take the training allowed multiple answers, and the participants were asked to select all applicable options. Data was tested for normality and homogeneity of variance; the assumption of normal distribution between pre-and post-scores was met (Shapiro-Wilk test p>0.05). A paired-samples t-test indicated that post-assessments scores were significantly higher (M = 64.17, SD = 5.41) than pre-assessments scores (M = 53.33, SD = 1.66), t(5) = 4.897, p = 0.004; Cohen’s *d* = 1.99. Knowledge changes per the test domains were analyzed; all domains except for “Follow up and maintenance” saw an increase in mean scores between pretest and posttest (Table 7). There was a significant increase in scores in the domains of “Prescription”, “User Training” and “Process” (Table 7).

**Table 6:** Characteristics of study population.

Characteristic	Hybrid Pittsburgh (n = 6)
Age, mean (SD)	26.33 (3.39)
Sex, Female, n (%)	5 (83.3)
Educational level, n (%)	
Bachelor	3 (50)
Graduate degree	3 (50)
Last educational training, n (%)	
Still attending	4 (66.7)
< 4 years	1 (16.7)
4 or more years	1 (16.7)
Employment status, n (%)	
20 hours/week	2 (33.3)
40 hours/week	4 (66.7)
Work setting, n (%)	
Academic	5 (83.3)
Outpatient	1 (16.7)
In-patient	1 (16.7)
Age group served, n (%)	
Early childhood	1 (16.7)
Adolescents	2 (33.3)
Adults	6 (100)
Older adults	4 (66.7)
Wheelchair services provision, Years, n (%)	
Less than 3 years	5 (83.3)
8 or more years	1 (16.7)
Previous wheelchair courses, n (%)	1 (16.7)
Service to wheeled mobility, Hours, n (%)	
Less than 3 hours	4 (66.7)
3–20 hours	2 (33.3)
Motivation for training, n (%)	
Professional growth	6 (100)
Personal growth	4 (66.7)
Member of an organization*, n (%)	4 (66.7)

SD: Standard deviation

*Organization providing wheelchair services

**Table 7:** Pretest and posttest scores of participants.

Test	Total Questions^†^	Pretest (n = 6)		Posttest (n = 6)		p-value
		Mean	Standard	Mean	Standard Deviation	
Domains						
Assessment	19	15.33	1.86	17.17	1.33	0.15
Prescription	12	7.5	0.84	11	0.63	*<0.0001
Fitting	10	4.5	1.05	5.5	2.74	0.482
Production	5	3.17	1.17	4.17	0.75	0.076
User’s Training	15	10.5	2.17	13.33	1.21	*0.016
Process	10	8.67	0.82	9.5	0.55	*0.042
Follow up and maintenance	4	3.67	0.52	3.5	0.84	0.741
Total scores	75	53.33	1.63	64.17	5.42	*0.004

*paired t-test significant at the <0.05 level

^†^ Each question values one-point

### Specific aim 3: Evaluate the quality of the Hybrid Course using the Quality Matters Higher Education Rubric

The evaluators’ reviews are included in Table 2. The rated rubrics from evaluators 1 and 3 met the QM standards by (1) reporting a mean total score above the 85%; and (2) scoring as “met” all essential standards. Nevertheless, results from evaluator 2 indicated that the total score did not fulfill the 85% threshold nor were essential standards were met; in particular, the essential standards not met were 2.4, 3.3 and 6.2. The percentage of agreement between evaluators in the QM rubric was 84% (Table 8).

**Table 8:** Evaluators’ percentage of agreement.

Specific Standard	Eval1	Eval2	Eval3	Eval1/Eval2	Eval1/Eval3	Eval2/Eval3	Agreement
1.1	3	3	3	1	1	1	1.00
1.2	3	3	3	1	1	1	1.00
1.3	2	0	2	0	1	0	0.33
1.4	2	2	2	1	1	1	1.00
1.5	2	2	2	1	1	1	1.00
1.6	1	1	1	1	1	1	1.00
1.7	1	1	1	1	1	1	1.00
1.8	1	1	1	1	1	1	1.00
1.9	1	1	1	1	1	1	1.00
2.1	3	3	3	1	1	1	1.00
2.2	3	3	3	1	1	1	1.00
2.3	3	3	3	1	1	1	1.00
2.4	3	0	3	0	1	0	0.33
2.5	3	3	3	1	1	1	1.00
3.1	3	3	3	1	1	1	1.00
3.2	3	3	3	1	1	1	1.00
3.3	3	0	3	0	1	0	0.33
3.4	2	2	2	1	1	1	1.00
3.5	2	2	2	1	1	1	1.00
4.1	3	3	3	1	1	1	1.00
4.2	3	3	3	1	1	1	1.00
4.3	2	2	2	1	1	1	1.00
4.4	2	2	2	1	1	1	1.00
4.5	2	2	0	1	0	0	0.33
4.6	1	0	1	0	1	0	0.33
5.1	3	3	3	1	1	1	1.00
5.2	3	3	3	1	1	1	1.00
5.3	3	3	3	1	1	1	1.00
5.4	2	2	0	1	0	0	0.33
6.1	3	3	3	1	1	1	1.00
6.2	3	0	3	0	1	0	0.33
6.3	2	2	2	1	1	1	1.00
6.4	1	1	1	1	1	1	1.00
6.5	1	1	1	1	1	1	1.00
7.1	3	3	3	1	1	1	1.00
7.2	3	3	3	1	1	1	1.00
7.3	2	2	0	1	0	0	0.33
7.4	1	0	0	0	0	1	0.33
8.1	3	3	3	1	1	1	1.00
8.2	3	3	3	1	1	1	1.00
8.3	2	2	2	1	1	1	1.00
8.4	2	0	2	0	1	0	0.33
8.5	2	2	2	1	1	1	1.00
**Total**	99	84	92				0.84

Eval: Evaluator

"1": Agreement

"0": Disagreement

The items considered problematic, those rated as “not met” by two or more reviewers were: *7*.*4 Course instructions articulate or link to an explanation of how the institution’s student services and resources can help learners succeed and how learners can obtain them*; and *8*.*6 The course was developed considering low bandwidth requirements*.

## Discussion

We developed a Hybrid Course based on the WHO WSTP-B using a systematic approach guided by an international committee of experts, the HSC, with experience delivering wheelchair training and developing educational programs for international contexts. The HSC was composed of members from low to high-income countries with experience developing and facilitating wheelchair service training in different settings. Studies have argued that stakeholder engagement is a key mechanism for increasing the relevance of research, promoting knowledge translation, and enhancing positive effects in a community [53–55]. Collaborating with stakeholders results in more usable, relevant and transferable knowledge that could help solve global health problems[54–56]. In particular, the rehabilitation field calls for a greater involvement and collaboration of stakeholders in all phases of the research process[54, 57–59] and we followed this guidance and ensured that the HSC was involved in all phases of the development and implementation processes.

In addition to our primary evaluation mechanism (international stakeholder feedback), we used the Quality Matters Standards to identify areas of opportunity for future development and provide insight into any areas that may be problematic for the initial implementation of the course. The Hybrid Course met the Quality Matters Standards in most of its reviews [29, 44] and reported a percentage of agreement between evaluators at an acceptable value of 84% (>80%) [46]. The two items considered problematic were *7*.*4 Course instructions articulate or link to an explanation of how the institution’s student services and resources can help learners succeed and how learners can obtain them*; and *8*.*6 The course was developed considering low bandwidth requirements*. A first approach to address these items could be (7.4) to clearly enlist the resources and support that the participants could access throughout the course; and (8.6) to state that the course was developed considering low bandwidth requirements. Given the fact that the Hybrid Course was developed considering web design guidelines for low bandwidth [41] and using an authoring tool and a learning management system that met low bandwidth requirements [50, 51] as described in the Methods and Results section, we believe that this item was marked as “not met” due to the lack of a clear statement of this characteristic and/or the inability of the reviewers to test this feature. The objective of this first quality review was to collect preliminary results that could help identify areas of opportunity. As the Hybrid Course is implemented in other settings, future studies could continue exploring the quality of the course and include additional sources of feedback such as the participants’ levels of satisfaction after the training intervention.

It is worth noting that multiple studies have used the QM Rubrics to guide the development of online and blended courses; however, the courses represented in those studies were developed for learners from the United States and other high-income countries [33–37]. In contrast, the Hybrid Course is intended for international contexts including low to high resource settings. Therefore, the HSC slightly adapted the QM Higher Education Rubric to include items that make it more contextually appropriate for less-resourced settings (e.g. *8*.*6 The course was developed considering low bandwidth requirements*, and *8*.*8 The course is hosted in a platform that allows mobile content access*). Future studies could continue working on this rubric and include validity evidence that support its use.

Additionally, there were significant increases in both the total ISWP Wheelchair Service Provision–Basic Test score and several domain scores, further demonstrating the training’s potential value and the feasibility of improving the participants’ basic wheelchair knowledge. Prior to the course, the participants’ mean pre-test score was slightly above the passing cutoff of the test (53 points), which indicates that our sample had some basic level wheelchair knowledge; despite that, the Hybrid Course still positively impacted the participants’ knowledge, reporting a significant increase in the mean post-test scores (p = 0.004) with an effect size (*d* = 1.99) that exceeded Cohen’s convention for large effect size (d = 0.80)[43]. This may suggest that the Hybrid has potential to increase knowledge for participants who have a range of baseline knowledge, including those who may be more advanced.

Moreover, paired t-tests were calculated per domain to explore specific knowledge change. Participants’ scores significantly increased after the training in some domains, but not all. There is some evidence to suggest that may be due to the emphasis placed on domain content through practical sessions in the in-person training and the participation of wheelchair users. For example, the "Prescription", "User Training" and "Process" domains all resulted in significantly higher scores, and the participants were able to fully experience those aspects of the provisioning process with wheelchair users in the practical sessions of the training. It is worth noting these domains also are highly represented in the test, suggesting that a significant increase would be more likely. However, this finding was not universal as the "Assessment" and "Fitting" domains, which are also highly represented in the test and include in-person practical sessions, did not demonstrate a significant increase on the post-test. This could be because the "Assessment" domain baseline knowledge was already high before the test (pre-score of 80.7%) and resulted in a final increase to 90.4%. It is important to note that the alpha level for multiple testing was not adjusted because this is an exploratory study with a small sample size and we were concerned about the potential of making a Type II error [60–62]. Furthermore, we were interested in exploring the magnitude of the effect rather than statistical significance. A larger sample size would help to determine the Hybrid Course’s specific impact on each domain and to explore which learning modalities are likely to have the biggest impact on the participants’ knowledge.

The Hybrid Course, due to its proposed scalability, flexibility, and effectiveness in improving participant knowledge, may have the potential to build local and global capacity by increasing the number of people trained in basic level wheelchair delivery. Increasing the number of trained personnel delivering wheelchair services may reduce secondary complications due to inappropriate wheelchair provision such as pressure injuries, postural deformities, and restricted breathing that may lead to better health outcomes among wheelchair users. The unique characteristics of the Hybrid Course design, such as its low bandwidth design [41], its mobile accessibility [47, 63], and its compatibility with SCORM and LMS [50, 51] allow the course to be adapted to different contexts and to be scaled to different levels [64, 65]. At the local level, governments could use the Hybrid Course to spread trainings across regions and to fulfill the promise of the United Nations Convention on the Rights of People with Disabilities. At the international level, the Hybrid Course could be an available tool for nations aligned with the WHO Global Cooperation on Assistive Technology (GATE) goals of promoting access to high-quality, affordable assistive products to lead a healthy, productive and dignified life [66]. Moreover, ISWP’s range of partners that including NGO’s, universities, and disability organizations [67] could use the Hybrid Course to implement trainings more efficiently. The reduced number of the in-person sessions and their associated costs, without compromising the effectiveness of the training, may allow organizations to allocate resources to more underserved regions.

Several limitations of this study are important to note in planning for future research and interpreting the current results. Although we have previously discussed the benefits of stakeholders’ engagement throughout all phases of the research process, the Hybrid Course may still face operational challenges that were not identified by our stakeholders’ group or were unsuccessfully addressed in the online development. These challenges would be realized more clearly through the implementation of the Hybrid Course in different contexts; this is part of our ongoing work. In this study, we had a small and highly educated sample size that may not reflect the population we intend to target and make our findings not generalizable to wheelchair providers from different settings.

We used the QM Higher Education Rubric to evaluate the quality of the Hybrid Course. This rubric uses a unidimensional approach with a dichotomous rating that asked reviewers to select if each of the Specific Standards had been “met” or “not met”. This approach may be too constricting and limit the analysis. A different scale format, like a 5-point Likert type-scale, could help quantify evaluators’ opinions and perceptions and provide detailed feedback on how to address the course’s limitations. Moreover, we only recruited three external reviewers that were not certified as Quality Matters’ Master Reviewers. Their responses were analyzed using a percentage of agreement that does not account for the impact of chance agreement and may overestimate or underestimate the agreement between evaluators [45, 46]. Conducting an official Quality Matters review or having more reviewers from different geographic locations may have uncovered greater disagreement between the evaluators or operational challenges in accessing the online modules. The implementation of the Hybrid Course in low-, medium- and high-income countries is a topic of ongoing work and will help us to address the limitations of this study and measure the impact of the training in other contexts.

### Ongoing & future work

Ongoing and future work will be focused on implementing the Hybrid Course in different contexts to evaluate the feasibility of the training program to be used for global capacity building. Implementation of the Hybrid Course was recently completed in India and Mexico (after translation into Spanish), and results are being compared to the traditional 5-day in-person training. The results of these comparisons will be the topic of future research publications. Additional future work includes translating the Hybrid Course into different languages, disseminating the training material through global partners, and expanding the Hybrid Course training program by moving more content into online training modules, including content from other WHO WSTP programs (e.g., the intermediate, managers’, stakeholders’, and trainers’ packages).

## Conclusions

A Hybrid Course on Wheelchair Service Provision for wheelchair providers in international contexts was developed using a systematic approach guided by an international group of stakeholders. The Hybrid Course met quality standards in two out of three evaluations and proved to be effective in increasing basic level wheelchair knowledge in a pilot study held at the University of Pittsburgh. Comparisons between the Hybrid and traditional in-person training are currently being performed as part of a feasibility study in international contexts.

## Supporting information

S1 File Dataset. (XLSX)
